# Renal Allograft Nephrectomy: Comparison Between Clinical and Pathological Diagnosis

**DOI:** 10.5812/numonthly.10596

**Published:** 2013-11-13

**Authors:** Ali Panahi, Reza Bidaki, Seyyed Mohammad Mahdy Mirhosseini, Darab Mehraban

**Affiliations:** 1 Department of Urology, Rafsanjan University of Medical Sciences, Rafsanjan, IR Iran; 2 Department of Psychiatry, Rafsanjan University of Medical Sciences, Rafsanjan, IR Iran; 3 Isfahan University of Medical Sciences, Isfahan, IR Iran; 4 Department of Urology, Tehran University of Medical Sciences, Tehran, IR Iran

**Keywords:** Kidney Failure, Chronic, Clinical Laboratory Techniques, Transplantation, Homologous

## Abstract

**Background and Aim:**

The most common complication of renal transplantation is allograft dysfunction, which in some cases leads to graft loss. The role of graft nephrectomy in the management of transplant failure is controversial. The procedure remains associated with a significant morbidity and also mortality. Our main purpose was the comparison between clinical and pathological diagnosis of graft nephrectomy.

**Patients and Methods:**

The documents of 88 patients who admitted for graft nephrectomy in Shariaty hospital for the last 25 years were reviewed. Slides of graft pathology were revised by an individual nephropathologist. Data was analyzed by SPSS 18 using ANOVA and Chi-square tests.

**Results:**

The percentages of clinical diagnoses for the graft nephrectomy are: chronic rejection (38%), graft infection (26%), gross hematuria (10%), acute rejection (10%), accelerated rejection (8%), hyper-acute rejection (4%) and thrombosis of the renal artery (4). On the other hand, the pathological diagnoses are: necrosis concomitant with thrombosis (35%), only necrosis (26%) and 5 (3) concomitant with 4 (3) in 16% of cases that means severe interstitial atrophy and fibrosis adjacent with acute cellular rejection and intramural vasculitis.

**Conclusions:**

Pathology included necrosis in about half of the graft nephrectomized patients. If the panel reactivity test is negative preoperatively, and there is no absolute indication for the operation, one may abstain from graft nephrectomy to save the patient, the morbidity and even the mortality of the procedure. On the other hand, the advantages of leaving the graft in situ are erythropoietin production, hydroxylation of calcidiol and maintenance of some residual diuresis.

## 1. Introduction

The most common etiology for allograft failure after the first year is an incompletely understood clinicopathological component variously named chronic rejection, transplant nephropathy, chronic renal allograft dysfunction, transplant glomerulopathy or chronic renal allograft nephropathy (1, 2). The last version of the revised Banff classification system has renewed as chronic allograft nephropathy, "interstitial fibrosis and tubular atrophy, without evidence of specific etiology" (3). The incidence of chronic kidney allograft nephropathy isn't known exactly, because of no universally accepted diagnostic criteria for this disorder. Generally, it is a poorly understood process that is defined as renal allograft dysfunction (occurring at least three months post-transplant) in the absence of active acute rejection, drug toxicity (principally calcineurin inhibitors), or other diseases. There are also diagnostic features on biopsy. The clinical diagnosis is suggested by gradual deterioration of graft function as manifested by slowly elevating plasma creatinine levels, increasing proteinuria (occasionally causing nephrotic range proteinuria), and worsening hypertension (HTN) (4-6). However, the reliance on these clinical features commonly results in the late identification of chronic renal allograft nephropathy, frequently culminating in allograft loss (7). Some of the risk factors have been identified for lower one-year deceased donor renal allograft survival, including second or third transplant, prior sensitization with more than 50 % panel reactivity, the presence of delayed graft function (defined as the requirement for dialysis during the first week post transplantation), the frequency and severity of rejection episodes, donor age less than 5 or more than sixty years, more degrees of HLA mismatching, and allograft dysfunction at discharge (plasma creatinine level more than 2 mg/dL (176 mol/L) (3). The etiology of kidney allograft dysfunction differs with the time post transplantation. Finally, the differential diagnosis is best approached by considering the time periods separately. The widely perceived success of transplantation must be tempered by the realization that organ demand far exceeds organ supply (8, 9). Furthermore, in spite of significant improvements in one-year graft survival, after the first year, the rate of chronic graft loss remains substantial. A European study has evaluated the determinants of survival post renal transplantation among 86 living donor transplant recipients and 916 cadaver donor recipients (7). After one-year post transplantation, an increased risk of death was observed among patients over the age of 40, men, cadaveric donor recipients, those with diabetes or hypertension, and smokers. Although transplantation confers the highest survival benefit among all the different renal replacement therapies, renal allograft recipients still have a high mortality rate compared with population controls. Our study will review the data relating to patient survival in patients undergoing renal transplantation.

## 2. Patients and Methods

With a look up to the pathology ward of the Shariaty hospital all files of the patients underwent graft nephrectomy from 25 years ago were extracted. All the document of 88 graft nephrectomy patients who admitted to this hospital in the past 25 years were gathered. Incomplete documents were excluded. Then pathological slides were obtained and matched with Banff classification 2007 with a single nephropathologist. Thereafter, admission files were reviewed and clinical, radiological and laboratory reasons for graft nephrectomy were extracted and gathered using SPSS software version 18 and analyzed by ANOVA and chi-square tests. Differences with the P-value < 0.05 was considered significant.

## 3. Results

From 88 files, only 80 files were completed and slides for revision were available. Mean age was 41-year-old with the range of 9-59 years. 57% of patients were male and 43% were female. Duration of the renal transplantation to graft nephrectomy between males and females was not statistically significant (P-value = 0.9) (Table 1). 

**Table1.** Demographic and Laboratory Data

Variable	Prevalence, %
**Age, y**	
Under 18	8
18-35	13
35-50	16
Elder than 50	63
**ESRD reason**	
DM	24
HTN	23
GN	10
VUR	6
Stone disease	5
UTI	4
ADPKD	1
Other	21
**Interval of renal allograft to nephrectomy**	
More than two years	60
Between 1 and 2 year	5
Between 3 months and 1 year	9
2 weeks to 3 months	12
Less than 2 weeks	14
**Creatinine serum level after transplantation**	
Equal or less than 1.5 mg/dL	95
Under 1.9 mg/dL	5
**Creatinine serum level at the time of the graft nephrectomy**	
2-3 mg/dL	24
3-4	27
4-5	13
5-6	11
6-7	12
More than 7	13

34% of graft biopsies before graft nephrectomy were inconclusive and only 64% was conclusive for diagnosis by the pathologist. 82% of patients hadn’t any history of ATN at the time of admission after renal transplantation and only 18% of patients were received at least one steroid pulse therapy at this time. Results of color Doppler ultrasonography in 10% of patients were rejection, in 20% were ATN, in 24% renal artery stenosis, in 30% renal vein thrombosis and in 16% were renal artery thrombosis. Between male and female these diagnoses were not significant. Isotope renography in about half of the patients represented ATN and in the other patients represented rejection; there was no significant difference between male and female. Clinical diagnosis before graft nephrectomy in 38% of patients were chronic rejection, 26% graft sepsis, 10% gross hematuria, 10% acute rejection, 8% accelerated rejection, 4% hyper-acute rejection and in 4% were renal arterial thrombosis. No meaningful difference observed between male and female. After revision of pathological slides by nephropathologist and matching with Banff classification 2007, 35% of cases pathological diagnosis were necrosis concomitant with thrombosis, 26% only thrombosis and in 16% 5 (3) concomitant with 4 (3), in 6% were 4 (3) concomitant with 6 and 4% 4 (2b) concomitant with 6; other diagnoses with same prevalence (1.6%) were necrosis concomitant with 2 (3), 4 (3), 2 (3) concomitant with 6, 6, ATN concomitant with thrombosis and 4 (3), 5 (3) concomitant with 4 (2b), 5 (3) concomitant with 2 (3) and in one patient pathology was normal. These diagnoses were not noticeable between male and female. In patients who underwent graft nephrectomy before two weeks most prevalent clinical diagnoses were hyper-acute rejection (46%), accelerated rejection (36%) and renal arterial thrombosis (18%). About patients who underwent graft nephrectomy between two weeks to three months most prevalent clinical diagnoses were acute rejection (50%), accelerated rejection (20%) and gross hematuria (20%), and for patients who underwent graft nephrectomy between three months to a year were acute rejection (43%), chronic rejection (29%) and gross hematuria (14%), for graft nephrectomy between 1-2 years these diagnoses were graft sepsis (50%) and chronic rejection (25%) and for more than two years were chronic rejection (50%), graft sepsis (29%) and gross hematuria (13%).

The most prevalent pathological diagnosis before two weeks was necrosis concomitant with thrombosis (64%), between two weeks to three months were 5 (3) concomitant with 4 (3) (30%), between three months to a year was necrosis concomitant with thrombosis (43%), between one year to two year was necrosis concomitant with thrombosis (50%) and after two years was necrosis (30%) and necrosis concomitant with thrombosis (29%).

**Figure 1.**
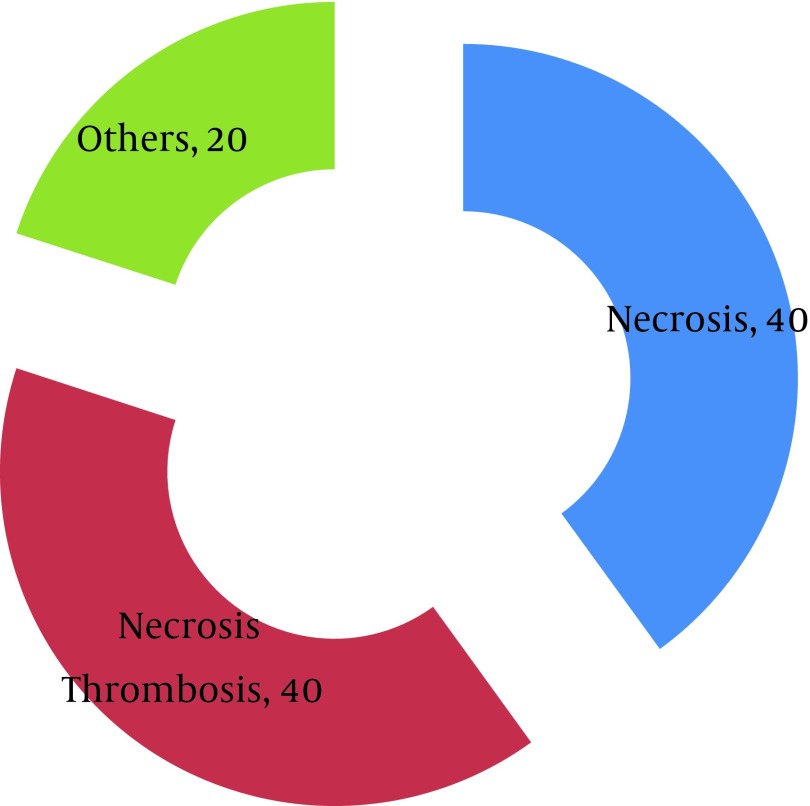
Final Pathological Diagnosis as Gross Hematuria

**Figure 2.**
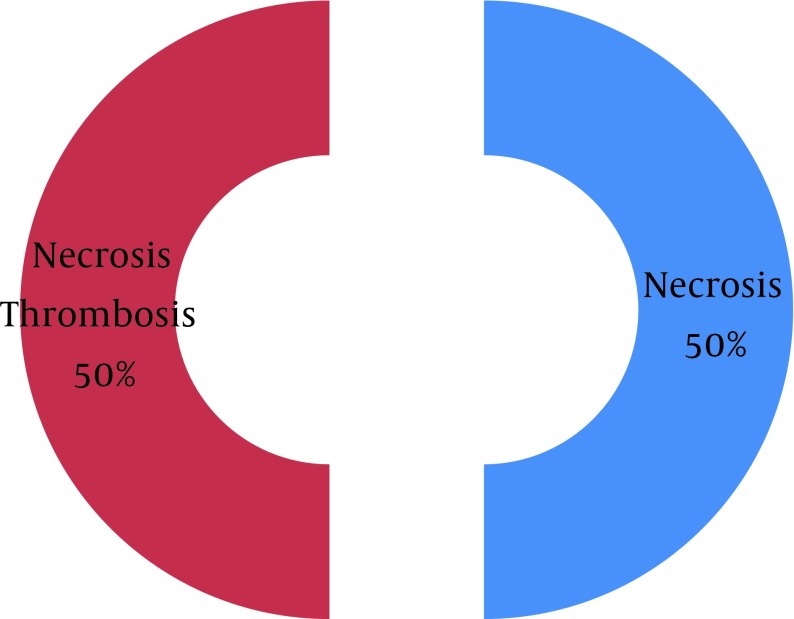
Final Pathological Diagnosis as Renal Arterial Thrombosis

In 60% of cases that clinical diagnoses were hyper-acute rejection, pathological diagnosis was necrosis concomitant with thrombosis, in 20% were 5 (3) concomitant with 4 (3) and in 20% was 4 (3). In 50% of cases that clinical diagnosis was accelerated rejection, pathological diagnosis was necrosis concomitant with thrombosis, in 33% was 4 (3) concomitant with 6 and in 17% was 5 (3) concomitant with 4 (3). In 33% of cases that clinical diagnoses were acute rejection, pathological diagnosis was necrosis concomitant with thrombosis, in 33% was 4 (3) concomitant with 6 and in 17% was only necrosis. In 26% of cases that clinical diagnosis was chronic rejection, pathological diagnosis was necrosis concomitant with thrombosis and in 33% was 4 (3) concomitant with 5 (3) so in 32% of cases that clinical diagnosis was graft sepsis, pathological diagnosis was necrosis concomitant with thrombosis and in 32% was only necrosis. In 40% of cases that clinical diagnosis was gross hematuria, pathological diagnosis was necrosis concomitant with thrombosis and in 40% was only necrosis but in 50% of cases that clinical diagnosis was renal artery thrombosis, pathological diagnosis was necrosis concomitant with thrombosis and in 50% were only necrosis (Figures 1 and 2).

## 4. Discussion

In this study, 50% of selected patients were male and half of them were female. Prevalence of ESRD causes between those were similar to other ESRD patients in population. Most of the graft nephrectomies (60%) were performed after two years following successful kidney transplantation. Most common reasons for graft nephrectomy were chronic rejection, graft sepsis and gross hematuria that were similar to other similar studies. Regardless of inconclusiveness of about one third of graft biopsies before graft nephrectomy, these patients were candidate for this procedure. Comparison of clinical and pathological results (that matches with Banff 2007 criteria) showed that in cases which graft nephrectomy was performed under two weeks from transplantation the most common clinical diagnosis was hyper-acute rejection and the most prevalent pathological diagnosis was necrosis concomitant with thrombosis; that justified this so in cases which graft nephrectomy was performed two weeks to three months from transplantation the most prevalent clinical diagnosis was acute rejection and the most prevalent pathological diagnosis was 5 (3) concomitant with 4 (3) (interstitial atrophy and fibrosis concomitant with cellular rejection and intramural vasculitis); that justified this. Comparison in cases that graft nephrectomy was performed under three months to a year from transplantation the most prevalent clinical diagnosis was acute rejection and the most prevalent pathological diagnosis was necrosis concomitant with thrombosis and in cases that graft nephrectomy was performed under a year to two years from transplantation the most prevalent clinical diagnosis was graft sepsis and the most prevalent pathological diagnosis was necrosis concomitant with thrombosis and in after two years most prevalent clinical diagnosis was chronic rejection then graft sepsis and most prevalent pathology was necrosis that justified them. According to our results in high percentages of patients ultimate pathologic diagnosis expressed T-cell mediated or antibody mediated rejection and not graft necrosis; maybe with change of immunosuppressive drugs or use of more potent drugs prevents graft loss and graft nephrectomy. In other percentages of patients ultimate graft pathology was necrosis; the surgeon did not perform graft nephrectomy if the tissue did not have any immunologic reactivity in recipient body (with check panel reactivity test), patient had not any symptoms and immunosuppressive drugs in the lowest level. In this category, patients could gain from advantages of leaving graft in situ. Zargar showed that frequency of graft nephrectomy was 4.8% (10) and in our study was 4%; in other reports this was 0.5-44%. Mean duration between transplantation and graft nephrectomy in his study was 5 years (10) and in our study was 3.5 years. In our study most of the graft nephrectomies were performed after 6m and in Zargar study this number was 65%. In his study pathological diagnosis was not correlated with Banff criteria thus comparison of these results was impossible. On the other hand, in 1995, Madore showed that need to graft nephrectomy was related to frequency of acute rejection episodes before it (11), but in our study this was not approved.

## 5. Conclusion

In some patient with kidney allograft loss, the graft could be salvageable with immunosuppressive therapy or other medical cares and surgeon can postpone and even revoke graft nephrectomy for the decline of morbidity and mortality. In other patients if graft asymptomatic and graft biopsy was shown necrosis, we can leave the graft in situ for use of its advantages.
